# Influence of Febrile Neutropenia Period on Plasma Viscosity at Malignancy

**DOI:** 10.1155/2013/507270

**Published:** 2013-10-27

**Authors:** Ibrahim Tek, Selami Kocak Toprak, Efe Hasdemir, Samed Rahatli, Aysegül Yesilkaya

**Affiliations:** ^1^Department of Medical Oncology, Medicana International Hospital Cancer Center, 06520 Ankara, Turkey; ^2^Department of Hematology, Baskent University School of Medicine, 06490 Ankara, Turkey; ^3^Department of Medical Oncology, Baskent University School of Medicine, 06490 Ankara, Turkey; ^4^Department of Infectious Diseases and Clinical Microbiology, Baskent University School of Medicine, 06490 Ankara, Turkey

## Abstract

Cancer, chemotherapy, and infections all together make changes in blood rheology and may affect the defense mechanisms by changing the thrombocyte function and endothelial cell. We have examined changes of blood rheology on plasma viscosity to put on probable following criteria for starting the treatment of febrile neutropenia immediately. A total of 27 postchemotherapy patients (16 males and 11 females) with febrile neutropenia diagnosed according to international guidelines have been included into the study. The plasma viscosity of the patients whose febrile neutropenia has been successfully treated was also measured to assess the impact of the duration of neutropenia on viscosity. The plasma viscosities of the patients were significantly higher during neutropenic episode than in nonneutropenic state (*P* = 0.006) except for alkaline phosphatase. All study parameters, particularly acute phase reactants, were statistically similar during both states. In the correlation of analysis with study parameters and stages, significant correlation was not observed between plasma viscosity alteration and leukocyte-neutrophil alteration, also other study parameters. We have demonstrated significantly elevated plasma viscosity in our patients during febrile neutropenic episode. Despite normal values of various parameters known to trigger plasma viscosity, particularly fibrinogen, it can be easily argued that the main mechanism may be the endothelial injury during infectious process and immune response mediated microcirculatory blood flow alterations.

## 1. Introduction

Infectious diseases are important factors of morbidity and mortality in patients with hematological malignancies. Even though cancer is a risk factor for infection, neutropenia has been regarded as the main factor for the development of infections in patients undergoing chemotherapy. Although there are so many new developments on diagnosis of infections and antimicrobial treatments, death caused by infections secondary to neutropenia is so common in acute and chronic leukemia patients. For the prevention of infection, early diagnosis and early treatment of infection become important in patients with cancer. Markers for febrile neutropenia are necessary for decision making of prophylaxis or medical treatment. 

Cancer, chemotherapy, and infections (all together) make changes in blood rheology and may affect the defense mechanisms by changing the thrombocyte function and endothelial cell [1]. Moreover, febrile neutropenia induced immune specific response could produce increased plasma viscosity, which leads to the hyperviscosity syndrome. Abnormalities in blood rheology might play an important role in the pathogenesis of organ failure in febrile neutropenic patients by damaging microvascular blood flow [2].

In this study, we have examined changes of blood rheology on plasma viscosity in order to start treatment of febrile neutropenia immediately.

## 2. Materials and Methods

### 2.1. Study Population

A total of 27 postchemotherapy patients (16 males and 11 females; mean age 63 ± 16.4) diagnosed with febrile neutropenia according to international guidelines have been included in our study (Table 1) [3]. All the patients were treated in the same clinic by the same physicians and healthcare staff, which increases the consistency of febrile neutropenia management. 

The patients had various types of malignancies. Seven of 27 patients had tumor of solid organ, 5 had palliative chemotherapy (two-non-small-cell lung cancer, primitive neuroectodermal tumor (PNET), two of Kaposi's sarcoma [KS]), and two had adjuvant chemotherapy (gastric cancer) (Table 2). The remaining patients had hematologic malignancies, most of which (*n* = 20) were acute myeloblastic leukemia (AML) patients who were receiving consolidation treatment after remission-induction therapy. Three patients were receiving remission-induction chemotherapy because of recently diagnosed acute lymphoblastic leukemia (ALL) (Table 2). In patients with ALL, lung cancer, PNET and KS the disease was not under control (8/27) (Table 1).

Most patients (78%) were treated with carbapenems (imipenem or meropenem), while six patients (22%) received piperacillin/tazobactam. Patients who failed empiric antibiotic therapy received vancomycin, and those with persistent fever (>5 days) received antifungal therapy (amphotericin B). All patients with prolonged neutropenia received hematopoietic growth factors such as granulocyte colony-stimulating factor (27/27). Success of antimicrobial therapy was defined as resolution of fever and clinical signs of infection, eradication of the microorganism without changing the empiric antibiotic regimen, and maintenance of the response after discontinuation of therapy.

The study protocol was approved and signed by the local ethics committee. Also all the participants were informed of the consent.

### 2.2. Methods

The plasma viscosity of the patients whose febrile neutropenia has been successfully treated, also measured to assess the impact of the duration of neutropenia on viscosity. The patients had febrile neutropenia during their clinical treatment (*n* = 17) and after being discharged (*n* = 10). The patients who were administered to outpatient clinic with febrile neutropenia had AML (*n* = 5), gastric cancer (*n* = 2), KS (*n* = 2), or PNET (*n* = 1) (Table 1). All patients were evaluated by an experienced physician according to Multinational Association for Supportive Care in Cancer (MASCC) scoring system [4, 5]. After taking blood and urine samples according to the international guidelines, empiric, broad-spectrum intravenous antibiotic therapy was administered until resolution or outcome of the event [6].

All tests were performed in the morning, after 8 hours of fasting. All subjects were seated for 5 minutes before collection. Tourniquets were used, and all collections were completed in less than 1 minute. Venous blood was collected into 5 mL ethylenediaminetetraacetic acid tube (anticoagulated tubes) for plasma viscosity analysis. All samples were frozen because immediate measurement of plasma viscosity was not possible. By this method, all samples were studied at the same time, and errors that could be due to calibration of the test machine were minimized. Blood samples taken into anticoagulated tubes were first centrifuged at 3000 rpm for 5 min, and then the separated plasma was frozen at −40°C. On the day of measurement, all samples were melted and recentrifuged (500 rpm for 2 min) and then measured at 37°C in a Brookfield DV-II+ Cone Plate Viscometer (Brookfield, Stoughton, MA, USA) machine, which was calibrated with distilled water (0.69 mPa.s.). Each sample was measured four times, and the average of the measurements was taken into account. Some sources indicate that the normal value of plasma viscosity is between 1.3 and 1.65 mPa.s., while others state that it is 1.10–1.30 mPa.s. at 37°C and independent of age and gender [7, 8].

### 2.3. Statistical Analysis

Statistical analyses were performed with SPSS software of Windows (Statistical Product and Service Solutions, version 15.0, SSPS Inc, Chicago, IL, USA). Analysis of variance was performed in order to examine the difference between the two groups with respect to viscosity and other laboratory parameters due to the presence of febrile neutropenic episode. Quantitative variables were expressed as mean values ± standard deviation (SD) for normally distributed data and compared with paired *t*-test. Analysis of variables with nonparametric distribution was performed by Wilcoxon signed-rank test. Spearman's rho test was applied for correlation analysis. 

## 3. Results

Data from 27 febrile neutropenic episodes were analyzed. The mean age was 63 ± 16.4 (range 31–87) years with 50% of patients 60 years or older (*P* = 1.000). 

Mean duration of febrile neutropenia development from the initiation of chemotherapy was 11.76 ± 2.5 days for 17 patients with AML, while it was 8.21 ± 1.1 days for 3 patients with ALL. This duration was 13.56 ± 0.7 days for the remaining 7 patients with solid organ tumors (*P* = 0.09). Mean duration of hematopoietic growth factor initiation was 7.32 ± 1.17 days for AML patients, 9th day for three ALL patients, and 5.8 ± 1.37 days for patients with solid organ cancer (*P* = 0.08).

MASCC risk index score was <21 in all patients (*n* = 27) (Table 1). Primary empiric carbapenem was administered to 11 AML patients, 3 ALL patients and 7 patients with cancer of solid organ. The remaining 6 AML patients received piperacillin/tazobactam (*P* = 0.07). In the following days, ALL patients who received remission-induction chemotherapy were started on vancomycin; also amphotericin B (*P* = 0.04) was added subsequently. Only vancomycin was added to the treatment of 4 AML patients, and 2 patients with KS (*P* = 0.04). 

Positive blood culture was obtained from only 7 AML patients, all of which were sensitive to the initiated antibiotherapy (Klebsiella pneumoniae and Pseudomonas aeruginosa; carbapenems).

There were no differences between duration of exiting febrile neutropenic episode with respect to diagnosis (*P* = 0.09).

The plasma viscosities of the patients were significantly higher during neutropenic episode than nonneutropenic state (*P* = 0.006). Except for alkaline phosphatase, all study parameters, particularly acute phase reactants, were statistically similar during both states (Table 3). Plasma viscosities were independent from age, sex, and diagnosis of the patients (*P* = 0.08). Moreover, when antibiotherapy was evaluated, plasma viscosities were similar in patients who received first line carbapenems or piperacillin/tazobactam and patients who were given vancomycin or vancomycin + amphotericin B in the following days (*P* = 0.09). Additionally, plasma viscosities of 7 patients with positive blood cultures were also similar to negative culture subjects (*P* = 0.08). 

The duration between chemotherapy initiation to development of febrile neutropenic episode, time to initiation of hematopoietic factor, and duration of exiting febrile neutropenic episodes were similar when compared to plasma viscosities (*P* = 0.07). There were no differences with respect to plasma viscosities with 8 patients who have uncontrolled disease, and also comparisons have been practiced to the remaining patient (*n* = 19) (*P* = 0.1). 

Similarly, plasma viscosities were similar in the outpatient (*n* = 10) and in the patient (*n* = 17) subjects (*P* = 0.09). Additionally, plasma viscosities were independent from chemotherapy regimens utilized (*P* = 0.06). 

In the correlation analysis with study parameters and stages, significant correlation was not observed between plasma viscosity alteration and leukocyte-neutrophil alteration.

When patients over and under 60 years old were analyzed separately, duration of febrile neutropenia development, time to initiation of hematopoietic growth factor, choice of first line antibiotherapy, response to antibiotics, and duration of exiting neutropenic episode were not statistically different with respect to plasma viscosities. 

## 4. Discussion

The relationship between hemorheological variables, especially plasma viscosity and febrile neutropenia, has not been extensively investigated. It has previously been reported that plasma viscosity is valuable and can be a surrogate marker of erythrocyte sedimentation rate and the other acute-phase reactants [8]. Nevertheless, the use of plasma viscosity in clinical practice has been ignored for many years. Plasma viscosity is a major determinant of blood flow in the microcirculation. Actually, plasma viscosity consists of water and blood macromolecules in protein structure. It is not affected from hematocrit value, red blood cell aggregation problems, and hemoglobinopathies, while inflammation and tissue damage affect plasma viscosity through alterations in plasma proteins with high sensitivity [9]. 

In our study, plasma viscosity values of the patients during febrile neutropenic episodes were significantly higher compared to values during exiting the neutropenic episode. Compared to other variables, only alkaline phosphatase (ALP) accompanied the white blood cell (WBC)/neutrophil difference between the two stages. Various acute phase reactants known to affect plasma viscosity such as C reactive protein (CRP), fibrinogen, and erythrocyte sedimentation rate (ESR) values were not different between both stages. CRP increases during a number of malignancies, connective tissue disorders and bacterial infections [9]. In our patients, CRP level was elevated during both stages; however, no significant difference was observed between the stages. In this instance, its effect on the plasma viscosity alterations in our population seems to be insignificant. Similarly, effect of ESR on the plasma viscosity appears to be insignificant as it is elevated in both stages, while the difference between the episodes is not statistically significant. 

Plasma viscosity is influenced by the concentration of plasma proteins and lipoproteins with the major contribution of fibrinogen [10]. Fibrinogen alterations are frequently observed, particularly during the course of hematologic malignancies. This can alter the whole blood viscosity by directly effecting aggregation properties of red blood cells. Interestingly, mean fibrinogen value of our patients was within normal values and similar in both stages. As a result, fibrinogen seemed to have no effect over plasma viscosity alterations. 

ALP is present in a number of tissues in the body and is particularly concentrated in liver, bile duct, kidney, and bone. Although tissue specific isoenzyme typing is not performed, it is obvious that elevated ALP values in patients who survived neutropenic episode is due to bone marrow activity. However, in the correlation analysis it was shown that ALP elevation was not accompanying viscosity alterations. 

In that point the main question is, as acute phase reactants and other main factors known to affect plasma viscosity are similar during both stages, why and by which mechanism is plasma viscosity significantly higher during febrile neutropenic episode compared to following stage? 

In this context, it is suggested that host defensive responses and modifications of blood properties are triggered in infectious process, and as a result, thrombocytes and endothelial functions are damaged—endothelial cells affected from infection are known to bind more thrombocytes—and blood flow in the microcirculation is decreased and diseased [11]. Moreover, it is known that in the presence of infection, in addition to erythrocyte redistribution, microvascular resistance is increased and blood viscous behaviour is modified in the capillary bed [1]. Actually, it can easily be said that plasma proteins are elevated during the infections, by the effects of both acute phase reactants and immune specific reactants which results in hyperviscosity. However, our study has interestingly shown that elevated plasma viscosity is triggered by a mechanism independent from acute phase reactants. In this situation, it should be kept in mind that leukocyte alterations, particularly red blood cell (RBC) volume and deformability, are also other important factors during hyperviscosity process. As RBC and WBC alterations are commonly observed during the natural course of hematologic malignities, plasma viscosity was studied instead of whole blood, as the former is not affected from these variables. 

The results of our study failed to demonstrate any differences with respect to electrolytes, kidney function tests and liver enzymes between two stages. Bilirubin and plasma protein values accompany these results in the same direction. Total protein and albumin values were low in both stages without significant difference. Inflammations and tissue injuries affect plasma viscosity by altering plasma protein levels. But the physicochemical or rheological approach states that the contribution of plasma proteins to plasma viscosity depends on their concentration, molecular weight, rigidity, and asymmetrical shape [9]. Hence, it does not mean that every plasma protein elevation will increase plasma viscosity as expected and also plasma viscosity may also be increased despite decreased plasma protein values. 

In conclusion, we demonstrated significantly elevated plasma viscosity in our patients during febrile neutropenic episode despite normal values of various parameters known to trigger plasma viscosity, particularly fibrinogen. It is suggested that the main mechanism may be the endothelial injury during infectious process and immune response mediated microcirculatory blood flow alterations. Although biochemical variables of this process are not studied, the absence of a study demonstrating the relationship between febrile neutropenia and plasma viscosity in the literature could permit such a speculation. In light of our presented data, it can be concluded that high plasma viscosity is a predictor of febrile neutropenic episode in patients with malignities. Further researches including larger and homogenous patient populations which will investigate microcirculatory mediator cytokines besides acute phase reactants will help to identify the relationship between febrile neutropenia and plasma viscosity.

## Figures and Tables

**Table 1:** Patient's demographic, clinic, and laboratory results.

Characteristics	*n*
Gender	
Male	16
Female	11
MASCC risk index	
High (<21)	27
Low (≥21)	0
Underlying cancer	
Hematological	20
Solid tumors	7
Patients status at presentation	
Inpatients	17
Outpatients	10
Burden of illness	
Mild signs	20
Severe signs	7
ECOG performance status	
0–2	20
3-4	7
Disease status	
Controlled	19
Uncontrolled	8
Treatment setting	
Induction (hematological)	3
Consolidation (hematological)	17
Locally advanced (solid tumors)	5
Adjuvant (solid tumors)	2
Infection documentation	
Microbiologically documented	7
Fever of unknown origin	20

MASCC: multinational association for supportive care in cancer; ECOG: eastern cooperative oncology group.

**Table 2:** According to diagnosis used chemotherapy regimens.

Diagnosis	Chemotherapy agents	*n*
AML	High dose cytarabine 3 gr/sqm q12h iv d1, 3, and 5	17
ALL	Remission induction chemotherapy^2^	3
Gastric cancer	5-Fluorouracil 680 mg/d; folinic acid 40 mg/d (iv d1–5)	2
Lung cancer^1^	Paclitaxel 250 mg/d; carboplatin 400 mg/d (iv d1)	2
PNET	Carboplatin 400 mg/d (iv d1); etoposide 140 mg/d (iv d1–3)	1
Kaposi's sarcoma	Vinblastine 8 mg/d (iv d1)	2

^1^Nonsmall cell.

^2^Cyclophosphamide 1000 mg/sqm/d iv d1, daunorubicin 45 mg/sqm/d iv d1-3, vincristine 2 mg/d iv d1, 8, 15, and 22, prednisolone 60 mg/sqm/d po d1-28, and L-asparaginase 6000 IU/sqm/d iv 6 doses.

AML: acute myeloblastic leukemia; ALL: acute lymphoblastic leukemia; PNET: primitive neuroectodermal tumor.

**Table 3:** Laboratory results of febrile neutropenic episode and after febrile neutropenic period.

Parameter	Febrile neutropenic episode	After febrile neutropenic period	*P*
	mean ± SD (median)	
Plasma viscosity (mPa.s.)	1.15 ± 0.08 (1.17)	1.07 ± 0.07 (1.07)	**.006**
Leukocyte (×10^9^/L)	0.93 ± 0.51 (0.9)	6.78 ± 4.03 (4.69)	**.001**
Neutrophil (×10^9^/L)	0.23 ± 0.22 (0.13)	3.88 ± 2.87 (2.54)	**.001**
Hemoglobin (gr/dL)	9.38 ± 1.63 (9.12)	9.67 ± 1.01 (9.63)	.177
Thrombocyte (×10^9^/L)	66.80 ± 60.95 (41.00)	94.77 ± 35.07 (63.95)	.245
ESR (mm/h)	34.21 ± 18.89 (33.00)	30.91 ± 12.01 (27.50)	.323
Prothrombin time (second)	14.76 ± 2.13 (14.25)	15.69 ± 2.86 (14.40)	.283
Fibrinogen (mg/dL)	379.53 ± 147.39 (352.00)	381.40 ± 120.15 (420.50)	.865
BUN (mg/dL)	18.97 ± 12.20 (15.50)	19.00 ± 11.60 (13.05)	.844
Creatinine (mg/dL)	0.82 ± 0.37 (0.74)	0.75 ± 0.36 (0.67)	.213
Sodium (mmol/L)	134.67 ± 3.88 (136.00)	138.73 ± 5.88 (139.00)	.136
Potassium (mmol/L)	3.86 ± 0.57 (3.89)	3.85 ± 0.43 (3.82)	.588
Uric acid (mg/dL)	5.33 ± 1.59 (5.33)	5.17 ± 1.29 (5.15)	.478
AST (U/L)	23.19 ± 13.13 (16.50)	25.81 ± 21.18 (16.10)	.846
ALT (U/L)	26.45 ± 23.95 (17.10)	26.18 ± 22.96 (16.20)	.476
ALP (IU/L)	100.83 ± 96.96 (63.20)	119.77 ± 48.15 (111.70)	**.031**
GGT (U/L)	104.12 ± 91.51 (33.50)	79.06 ± 78.00 (41.50)	.698
Total bilirubin (mg/dL)	1.12 ± 0.45 (1.08)	1.74 ± 0.97 (1.09)	.927
Direct bilirubin (mg/dL)	0.43 ± 0.18 (0.43)	1.08 ± 0.25 (0.55)	.167
Total protein (gr/dL)	5.12 ± 0.82 (5.20)	5.40 ± 0.44 (5.30)	.268
Albumin (gr/dL)	3.07 ± 0.50 (3.01)	3.03 ± 0.47 (3.20)	.934
C reactive protein (mg/L)	65.79 ± 63.02 (54.30)	71.53 ± 67.89 (48.60)	.948

ESR: erythrocyte sedimentation rate; BUN: blood urea nitrogen; AST: aspartate aminotransferase; ALT: alanine aminotransferase; ALP: alkaline phosphatase; GGT: gamma-glutamyl transferase.
